# Patient satisfaction with primary care: an observational study comparing anthroposophic and conventional care

**DOI:** 10.1186/1477-7525-6-74

**Published:** 2008-09-30

**Authors:** Barbara M Esch, Florica Marian, André Busato, Peter Heusser

**Affiliations:** 1 Doctoral candidate, University of Berne, Switzerland; 2 Department of Anthroposophic Medicine, Institute for Complementary Medicine KIKOM, University of Bern, Inselspital, 3010 Bern, Switzerland; 3 Institute for Evaluative Research in Orthopaedic Surgery, University of Bern, Stauffacherstrasse 78, 3014 Bern, Switzerland

## Abstract

**Background:**

This study is part of a cross-sectional evaluation of complementary medicine providers in primary care in Switzerland. It compares patient satisfaction with anthroposophic medicine (AM) and conventional medicine (CON).

**Methods:**

We collected baseline data on structural characteristics of the physicians and their practices and health status and demographics of the patients. Four weeks later patients assessed their satisfaction with the received treatment (five items, four point rating scale) and evaluated the praxis care (validated 23-item questionnaire, five point rating scale). 1946 adult patients of 71 CON and 32 AM primary care physicians participated.

**Results:**

1. Baseline characteristics: AM patients were more likely female (75.6% vs. 59.0%, p < 0.001) and had higher education (38.6% vs. 24.7%, p < 0.001). They suffered more often from chronic illnesses (52.8% vs. 46.2%, p = 0.015) and cancer (7.4% vs. 1.1%). AM consultations lasted on average 23,3 minutes (CON: 16,8 minutes, p < 0.001).

2. Satisfaction: More AM patients expressed a general treatment satisfaction (56.1% vs. 43.4%, p < 0.001) and saw their expectations completely fulfilled at follow-up (38.7% vs. 32.6%, p < 0.001). AM patients reported significantly fewer adverse side effects (9.3% vs. 15.4%, p = 0.003), and more other positive effects from treatment (31.7% vs. 17.1%, p < 0.001).

Europep: AM patients appreciated that their physicians listened to them (80.0% vs. 67.1%, p < 0.001), spent more time (76.5% vs. 61.7%, p < 0.001), had more interest in their personal situation (74.6% vs. 60.3%, p < 0.001), involved them more in decisions about their medical care (67.8% vs. 58.4%, p = 0.022), and made it easy to tell the physician about their problems (71.6% vs. 62.9%, p = 0.023). AM patients gave significantly better rating as to information and support (in 3 of 4 items p [less than or equal to] 0.044) and for thoroughness (70.4% vs. 56.5%, p < 0.001).

**Conclusion:**

AM patients were significantly more satisfied and rated their physicians as valuable partners in the treatment. This suggests that subject to certain limitations, AM therapy may be beneficial in primary care. To confirm this, more detailed qualitative studies would be necessary.

## Background

The modern view of quality of care looks to the degree to which health services meet patients' needs and expectations [1], both as to technical and interpersonal care [2]. Moreover, in times of a dramatically changing post-industrial knowledge-based society and in the context of finite budgets and increasing health care costs, it becomes more and more important to deliver medicine that meets the subjective needs of patients [3].

Evaluation of patient satisfaction is accepted as a valuable addition to other types of outcome measures (such as health status, quality of life or costs) in measuring the quality of general practice care [3,4].

The increased use of complementary and alternative medicine (CAM) in the Western world [5,6] has also resulted in a high demand for various CAM procedures in Switzerland. Several studies conducted over the past 20 years show that approximately half of the Swiss population uses and appreciates CAM; the same percentage (ca. 50%) of Swiss physicians believe CAM is effective. The majority (>50%) of the Swiss population prefer a CAM hospital to a CON hospital, and the vast majority (>85%) are in favour of basic health insurance reimbursing costs of CAM treatment [7]. About 10.6% of the Swiss population in 2002 utilized at least one of the five most important CAM methods (75% utilized CON and 33% all CAM methods) [8].

The high popularity and extensive use of CAM has resulted in inclusion of certain CAM methods in basic health insurance in several countries. In this context, in Switzerland the five most important CAM methods practiced by physicians, namely anthroposophic medicine (AM), homeopathy, neural therapy, phytotherapy and traditional Chinese medicine, were temporarily included in the basic compulsory health insurance scheme from 1998 to 2005. At the same time, additional research into the effectiveness and cost-benefits of CAM was initiated, such as the cross-sectional nationwide evaluation of primary care funded by the Swiss Federal Office of Public Health conducted between 1998 and 2005 (PEK: Programm Evaluation Komplementärmedizin, complementary medicine evaluation programme) [4], of which the present study is a part. The political debate on reimbursement of CAM treatment is ongoing.

PEK investigated among other CAM methods, AM, a physician-provided complementary therapy system that evolved from the work of Rudolf Steiner, PhD, Ita Wegman, MD, and other physicians since the 1920s. Conceptually, AM is based on the notion that the human being does not only consist of material energies, but also of specific forces of life, soul and spirit [9]. Thus, health, disease, and therapy effects do not result solely from molecular interactions, but also from differentiated causal interactions between these factors within the human being as a whole. Accordingly, additional therapeutic options at the levels of life forces, soul and spirit complement and integrate conventional treatments aiming at the physical level [9] by supporting organ functions, enhancing immune processes and balancing treatment side effects [10,11]. To do this, AM employs medicines derived from mineral or plant substances, counselling, art or music therapy, and therapeutic eurythmy, a movement therapy designed to establish harmony between functions of body, soul and spirit [9,12].

AM theory is compatible with the hermeneutic approach [13], which leads to understanding patients' individual points of view and their spiritual and existential questions [14,15]. AM emphasizes a close carer-patient relationship to support patients' coping efforts with disease [16,17], to give orientation, to enhance optimism and to engage patients in their own healing process in the sense of "salutary medicine" [18].

AM attemps to overcome the CONs body-soul dualism by seeing the autopoetic action of the soul in conjunction with the "life forces" for sustaining healthy and detrimental processes in the whole human being, which manifest themselves in psychological, physiological or organic processes [11]. AM therapy in this very broad sense acts even preventively and aimes neither unilaterally on the body nor unilaterally on the soul but treats the patient as a whole [9,19].

AM therapy has its principal application in treatment of patients with chronic diseases and in the treatment of children [20] and persistently improve disease symptoms and quality of life for chronically ill patients [21], and for patients with other illnesses, such as cancer [16,17].

An anthroposophic lifestyle (with restrictive use of antibiotics and antipyretics and a diet based on bio-dynamic and organic food) helps to prevent allergies in children [22].

In Switzerland, three state-approved AM hospitals, two departments in public hospitals, and one sanatorium offer AM treatment for over 200 in-patients. About 130 general practitioners deliver AM care to outpatients. AM physicians and hospitals provide the most popular holistic cancer treatment in Switzerland [17,23]. The Universities of Bern, Basel and Zürich offer courses in AM.

According to a meta-analysis on AM [20] 180 of 189 studies from European countries found positive effects from AM (better than no treatment; at least as good as CON treatment or, in studies without a control group, improvement of symptoms), yet methodological problems limit the validity of many of these studies.

Patient satisfaction with AM was very high, within the scope it was measured in these studies [20]. They show high treatment satisfaction with AM therapy for patients suffering from chronic diseases (asthma, depression, low back pain, migraine, and neck pain) [21] and acute ear infection [24], and a high satisfaction with the health status following AM therapy for patients with rheumatoid arthritis [25]. Finally, a degree of patient satisfaction can be presumed from higher life satisfaction [16] and compliance [23] and better quality of life and coping [17] resulting from AM therapy for cancer patients.

The results of a qualitative study in primary care suggest that AM patients were highly satisfied with the trustworthy personal care and support and the thorough technical care given by their physicians that differed from those they received in previous consultations with CON physicians. AM patients highlighted the holistic nature of the approach, its person-centeredness that was tailored to individual needs, its ability to look at underlying causes, the facilitation of personal learning and development, the use of natural treatments and remedies and the involvement of patients in the management of their illness [12].

Moreover, the Swiss-wide annual benchmarking and quality studies demonstrated very high levels of patient satisfaction in anthroposophic hospitals, particularly in respect of medical care, competence and communication skills [4,20].

The generally positive results of prior studies and the socio-economic und health policy issues set forth above have focussed attention on the place which CAM in general and AM in particular should have in the Swiss health system. Our study aims to present a realistic picture of physician-provided AM outpatient treatment of adult patients (> 16 years) in Switzerland with a wide range of diagnoses compared to a control group of patients from CON general practices and to evaluate the results in light of differences in structure (including theory), process and outcome between these groups.

## Methods

Patient satisfaction is a multidimensional concept, based on a relationship between experiences and expectations. The term patient satisfaction as used herein means the positive emotional reaction to the consultation and the positive experience of the treatment in its various aspects. Good communication [26], comprehensive assessment of patients' needs and provision of information [3], shared decision-making [27], supportive and well understanding physician-patient relationship, the physician's personal qualities [28], or simply positive treatment results for the patient, have all been shown to improve patient satisfaction. Many of the above factors are consistent with AM approach, which emphasises these concepts.

Patient satisfaction is difficult to distinguish from related concepts, such as "quality of life", "happiness" and "contentment" [29]. Under the view of the concept of quality of health care focusing on structure, process and outcome of care [1], patient satisfaction is part of the treatment result and at the same time a good indicator of quality of care [29]. In connection with the introduction of new therapy methods, patient satisfaction is investigated immediately after the exploration of effectiveness and costs [30]. In light of the increasing cost pressure in health-care systems patient satisfaction with primary care and the choice of therapy may also depend on the extent to which health care insurance reimburses the costs and whether and to what extent the patients have to bear these costs themselves.

Our data are based on two distinct parts of PEK study [4]. PEK evaluated health insurance expenditures for physicians employing the five CAM methods and tested patient satisfaction four weeks after the treatment compared with a control group of physicians providing conventional primary care (CON). The study included only certified CAM and CON physicians who were members of the Swiss Medical Association FMH.

In 2002, we collected data on the structure of primary care physicians and their practices (PEK I) with a mailed questionnaire. The questionnaire addressed physicians' age, gender, level of education, number of years since accreditation, part-time or full-time work, major language used, practice organization (group or solo practice; level of urbanization of practice location according to the classification of the Swiss Federal Statistical Office) and technical equipment (ECG, ultrasound, X-ray and laboratory).

In a second part of the study (PEK II), patients were questioned on their state of health, their treatment expectations and why they chose the treating physician. Separately, we asked physicians to specify the diagnosis, the seriousness of the illness, and treatment. Four weeks later, we mailed a follow-up questionnaire to the patients. Five items in this questionnaire were directed at patient satisfaction, side effects and fulfilment of expectations. The other 23 items were taken from a standardised international validated instrument for patients' evaluations of general practice care (Europep) [3].

### Physicians and patients

The inclusion criteria for physicians in the AM group were working as primary care provider for at least two days a week and membership in the Swiss Medical Association for Anthroposophic Medicine (VAOAS), which has the following prerequisites: Completed specialist training in a CON discipline, 360 hours of training in AM (as an assistant anthroposophic doctor in a clinic, practice, hospital department or independently together with a mentor), and participation in a study group of physicians for AM. Moreover we only included physicians, from whom we could sample at least five patients.

51 of the 134 members of the VAOAS, who were invited by letter, participated in the study. 32 met the inclusion-criteria of working as primary care provider for at least two days a week. In PEK II we matched 71 CON physicians who were not listed as members in any CAM medical association in Switzerland to AM physicians using a stratification technique based on geographic distribution. Three questionnaires evaluated structure, process and outcome of care.

Patients were classified according to the method of treatment they chose into the AM and CON groups. We only included patients over 16 who gave their written consent. The ethics committee of the Canton Berne raised no objection to the study. The study was conducted in compliance with the Helsinki Convention.

### Data collection

The structural data on the physicians and their practice were taken from PEK I. We developed the questionnaire in German, French and Italian together with an expert group of Swiss primary care providers specialized in CON and/or CAM.

Data collection took place in October 2002 and January, May and August 2003 on four different predetermined weekdays. Practice staff handed out a written questionnaire to all eligible patients consecutively visiting their practice on such days. Patients filled out the questionnaire in the waiting room prior to the consultation and returned it to the practice staff such that physicians were not aware of the content. The participating physicians were reimbursed with CHF 500 each.

Four weeks after, patients were sent a second questionnaire directed to the perceived effectiveness of, and their satisfaction with, the treatment, fulfilment of their expectations, and whether they experienced adverse or positive side effects or other effects as a result of the treatment. They were also sent the Europep instrument [3]. Europep evaluates medical care with 23 questions and a five-point answer scale ranging from poor to excellent. Six Europep questions addresses "doctor-patient relationship and communication", five questions addresses "medical-technical care", four questions addresses "information and support to patients", two questions addresses "continuity and cooperation", and six questions addresses "facilities, availability and accessibility".

### Data management and data analysis

All data were recorded using a relational database. Forms filled out by patients and physicians during consultations were coded and recorded manually. The questionnaires were machine-readable and were scanned by the Swiss Federal Office of Information Technology using Optical Character Recognition (OCR).

Data derived from the Europep questionnaire were reduced to a two-level scale with the most favourable answer category coded as one and all other non-missing categories as zero. These data were analyzed using hierarchical multivariate procedures for each individual question [31]. In addition to the AM group, patient age and gender were included in the models as additional factors. Similar models were used to evaluate the probabilities of complete symptom resolution, complete fulfilment of expectations and of being very satisfied with the treatment. All analytical procedures accounted for non-independence of observations at the practice level and 95% confidence intervals (95% CI) of means proportions and odds ratios were calculated accordingly.

The level of significance was set at p < 0.05 throughout the study and SAS 9.1 (SAS Institute Inc., Cary, NC, USA) was used for all calculations.

## Results

### Structural characteristics of physicians and their practices

The 71 CON and 32 AM physicians (see Table 1) did not differ significantly in age and clinical experience, but compared with CON more AM physicians were German speaking, female, worked part-time, in group practices, and in inner cities. Nearly all CON practices had a laboratory, ECG, X-ray and ultrasound, whereas most AM practices were only equipped with ultrasound. The consultations of AM physicians lasted on average seven minutes longer than those of CON physicians.

**Table 1:** Structural characteristics of physicians, practices and duration of visit (physician rated)

		**CON**			**AM**			**P-values**
			
		**#**	**%**	**CI**^c^	**#**	**%**	**CI**^c^	
**Physicians**	*Number*	71			32			
Female physicians*	*Proportion*	9	12.7		10	31.3		p = 0.025
**Age**	*Mean (Standard Deviation)*	52.3 (6.86)			51.4 (8.84)			P = 0.628
**Years since graduation**	*Mean (Standard Deviation)*	23.4 (7.40)			21.5 (9.19)			P = 0.301
**Language**	*German*	43	60.6		29	90.6		p = 0.008
	*French*	25	35.2		3	9.4		
	*Italian*	3	4.2		0	0		
Urbanisation*	*Inner city*	24	33.8		22	68.8		p = 0.004
	*Agglomeration*	35	49.3		7	21.9		
	*Rural area*	12	16.9		3	31.1		
**Practice type**	*Single practice*	51	71.8		17	53.1		P = 0.064
	*Group practice*	20	28.2		15	46.9		
**Level of activity**	*Full time*	64	91.4		24	77.4		p = 0.053
	*Part time*	6	8.6		7	22.6		
Practice equipment (*)^b^	Laboratory *	68	95.8		26	81.3		(p = 0.024)^b^
	ECG *	69	97.2		26	81.3		(p = 0.011)^b^
	X-ray *	57	80.3		9	28.1		(p < 0.001)^b^
	*Ultrasound*	16	22.5		7	21.9		(p = 1.000)^b^
Duration of Visit^a,*^	*Mean (min)*	16.8		15.7–18.0	23.3		21.1–25.9	p < 0.001

* = significant difference (p < 0.05) to COM-group in a multivariate logistic model,

^a ^= data from PEK II and (...)^b ^= Fisher's Exact Text

^c ^= 95% Confidence Interval

### Characteristics of patients and their expectations

Table 2 shows socio-demographic data, the self-rated health status of the participating patients, their reasons for consultation and their expectations. AM patients were primarily German speaking, female and better educated and more frequently reported chronic health problems than CON patients. Significantly more CON patients chose their physician for pragmatic reasons (for example, geographic proximity of the practice), whereas AM patients were more likely to choose their GPs based on the preferred procedure. The self-assessment of the patients of their illness in both groups was similar; however, AM patients had on average a higher risk of mortality, as measured by the Charlson index. Despite the higher risk of mortality, AM patients more frequently expressed the expectation of being healed.

**Table 2:** Demographic attributes, health status, expectations and reasons for seeking the physician

			**CON**			**AM**			**P-values **(X^2^-Test)
				
			**#**	**%**	**CI**^a^	**#**	**%**	**CI**^a^	
**Demographic attributes**	**Patients**	*Number*	1363	43.8		583	51.2		P = 0.005
	**Patient age**	*Mean (Standard Deviation)*	53.9 (17.21)			51.7 (16.75)			P = 0.104
	Female Patients*	*Proportion*	804	59.0		440	75.6		P < 0.001
	Language*	*German*	821	60.6		506	87.2		P < 0.001
		*French*	382	28.2		46	7.9		
		*Italian*	89	6.6		9	1.6		
		*Other*	63	4.6		19	3.3		
	Education*	*Proportion higher education*	330	24.7		223	38.6		P < 0.001
**Self rated health status**	**General health**	*Excellent*	63	4.7		19	3.3		P = 0.224
		*Very good*	269	20.2		102	17.8		
		*Good*	697	52.4		301	52.5		
		*Fair*	254	19.1		130	22.7		
		*Poor*	46	3.5		21	3.7		
	Chronic conditions*	*> 3 month*	630	46.2	43.5–48.9	308	52.8	47.4–58.3	P = 0.015
	**Severe conditions**		240	19.8	17.7–21.9	122	22.3	18.0–26.6	P = 0.332
**Expectations**	Healing*	*yes*	668	49.0		349	59.9		P < 0.001
		*no*	695	51.0		234	40.1		
	**Relief**	*Yes*	566	58.5		251	43.1		P = 0.438
		*no*	797	41.5		332	56.9		
Reasons for consultation*	**Pragmatic reasons**		743	64.7		101	17.9		P < 0.001
	**Quality of the physician**		381	33.1		186	33.0		
	**Preferred procedures**		25	2.2		277	49.1		

* = significant difference (p < 0.05) to CON-group in a multivariate logistic model

^a ^= 95% Confidence Interval

### Diagnosis and health status of the patients

The diagnosis of the patients in the two groups is shown in Table 3. There was a significant difference in the distribution of diagnoses between the two groups. AM patients were diagnosed more often with neoplastic diseases (ICD10 Codes C00-D48), whereas CON patients were twice as likely to have diseases of the circulatory system, injuries, poisoning and endocrine and metabolic diseases. With respect of the distribution of co-morbidity, there was no statistically significant difference between the groups (p = 0.398). Slightly more AM patients (65.01%) had two or more diagnosis as compared to 60.67% for the CON group. AM patients had significantly (p < 0.000) higher scores in the Charlson co-morbidity index [32], which indicates that they had higher mortality risks.

**Table 3:** Diagnoses, co-morbidities and Charlson index (physician rated)

**Main Diagnoses,** ICD-10* ^(Distribution p < 0.001)^		**CON**			**AM**		
		**#**	**%**	**CI**^a^	**#**	**%**	**CI**^a^
***M***	*Diseases of the musculoskeletal system*	238	17.46	14.9–20.0	111	19.04	15.3–22.8
***I***	*Diseases of the circulatory system*	241	17.68	15.4–19.9	51	8.75	6.1–11.4
***J***	*Diseases of the respiratory system*	135	9.90	8.3–11.6	65	11.15	8.5–13.8
***F***	*Mental and behavioural disorders*	112	8.22	6.1–10.3	63	10.81	7.5–14.2
***S. T***	*Injury, poisoning*	104	7.63	5.8–9.5	28	4.80	3.2–6.4
***K***	*Diseases of the digestive system*	86	6.31	4.8–7.8	42	7.20	5.0–9.4
***G, H***	*Diseases of the nervous system, eye and ear*	69	5.06	0.2–3.5	33	5.66	0.3–4.1
***E***	*Endocrine, nutritional and metabolic diseases*	79	5.80	4.5–7.0	15	2.57	1.2–3.9
***L***	*Diseases of the skin*	47	3.45	2.4–4.5	25	4.29	2.7–5.9
***N***	*Diseases of the genitourinary system*	42	3.08	1.9–4.3	29	4.97	2.9–7.0
***C,****D*1	*Neoplasms*	15	1.10	1.4–2.9	43	7.38	5.5–12.0
***A, B***	*Infectious and parasitic diseases*	23	1.69	1.0–2.3	14	2.40	1.1–3.7
***D2***	*Diseases of the blood*	20	1.47	0.1–0.8	13	2.23	0.1–1.6
***Z***	*Factors influencing health status and contact with health services*	71	5.21		12	2.06	
	*Others and not elsewhere classified diseases*	81	2.35		39	6.58	

**Co-Morbidity**^(p = 0.398)^							

	**None**	536	39.3		204	35.0	
	***1***	404	29.6		175	30.0	
	***>1***	423	31.0		204	35.0	

Charlson Index*^(p < 0.001)^							

	***0***	1207	86.6		509	84.5	
	***1***	115	6.8		21	2.2	
	***> 1***	41	1.9		53	6.2	

* = significant difference (p < 0.05) to CON-group in a multivariate logistic model

^a ^= 95% Confidence Interval

### Return rate of the questionnaires

1946 patients of 103 AM and CON GPs were evaluated, representing a proportion of returned questionnaires of 45.8% of the 4249 patients. 51.2%, of the AM patients responded as compared to 43.8% of the CON patients. Altogether, more females (49.8%) than males (40.9%) and more chronically ill (50.5%) than non-chronically ill patients (42.2%), responded to the survey. Responders were on average 53.3 years old, non-responders 9 years younger.

### Results of our questionnaires

As shown in Table 4, 56.1% of patients receiving AM treatment from their GP were significantly more satisfied with the overall treatment as compared to 43.4% in the CON group. 38.7% of the AM patients reported that the treatment completely fulfilled their expectations (vs. 32.6% for the GPs using CON). AM patients reported significantly fewer adverse side effects (9.3% for AM v.15.4% for CON). In 31.7% (vs. 17.1% in the group treated with CON) patients noted other positive effects and patients receiving AM treatment only complained of other negative effects in 3.0% of the responses (vs. 6.8% for the patients of GPs employing CON).

**Table 4:** Results of the questionnaire on patient satisfaction, fulfilment of expectations and side effects

		**CON**			**AM**			**X^2^-Test**
			
		**#**	**%**	**CI**^a^	**#**	**%**	**CI**^a^	
Overall Satisfaction*	*Proportion of "very satisfied"*	549	43.4	40.4 – 46.4	315	56.1	50.9 – 61.2	P < 0.001
Fulfilment of treatment expectations*	*Proportion of "complete fulfilled"*	409	32.6	29.2 – 35.9	212	38.7	33.5 – 43.9	P < 0.001
Adverse side effects? *	*Yes*	194	15.4	13.0 – 17.7	52	9.3	6.5 – 12.0	P = 0.003
Other effects? *	*Positive*	208	17.1	14.8 – 19.4	170	31.7	25.6 – 37.8	P < 0.001
	*Negative*	83	6.8	5.6 – 8.0	16	3.0	1.6 – 4.4	P < 0.001

* = significant difference (p < 0.05) to CON-group in a multivariate logistic model (age and gender controlled)

^a ^= 95% Confidence Interval

The characteristics of better satisfaction and higher likelihood of successful treatment as well as the absence of negative side effects were independent of age and gender of the patients.

Table 5 sets forth the percentage of the patients who gave the highest rating ("excellent") in the Europep instrument 4 weeks after their visit. AM patients valued their relationship and communication with their physicians more than did CON patients. As to the factors whether physicians make them feel they had time during the consultation (76.5% vs. 61.7%, p < 0.001), physicians' interest in the personal situation of the patients (74.6% vs. 60.3%, p < 0.001) and that the physician was listening to them (80.0% vs. 67.1%, p < 0.001), differences between the AM and CON group were highly significant. AM patients evaluated significantly more often that their physician made it easy for them to tell him or her about their problem (71.6% vs. 62.9%, p = 0.023) and that the physician involved them in decisions about their medical care (67.8% vs. 58.4%, p = 0.023).

**Table 5:** Patients rating their satisfaction as „excellent“ in the EUROPEP questionnaire four weeks after the consultation

**Questions/items**	**CON**		**AM**		**X^2^-Test**
		
	**%**	**CI**^a^	**%**	**CI**^a^	
**Relationship and communication**					
1. Making you feel you had time during consultation? *	61.7	57.9 – 65.4	76.5	72.1 – 80.9	P < 0.001
2. Interest in your personal situation? *	60.3	57.1 – 63.5	74.6	68.7 – 80.4	P < 0.001
3. Making it easy for you to tell him or her about your problem?*	62.9	59.0 – 66.9	71.6	65.6 – 77.6	P = 0.023
4. Involving you in decisions about your medical care? *	58.4	54.7 – 62.2	67.8	62.7 – 72.9	P = 0.022
5. Listening to you?*	67.1	64.1 – 70.1	80.0	75.8 – 84.3	P < 0.001
6. Keeping your records and data confidential? *	75.4	72.7 – 78.0	85.0	79.4 – 90.7	P = 0.002

**Medical care**					
7. *Quick relief of your symptoms?*	27.6	24.8 – 30.5	26.7	22.5 – 31.0	n.s.
8. *Helping you to feel well so that you can perform your normal daily activities?*	41.2	38.2 – 44.3	45.4	39.9 – 50.9	n.s.
9. Thoroughness? *	56.5	52.9 – 60.1	70.4	64.3 – 76.5	P < 0.001
10. *Physical examination of you?*	52.6	49.7 – 55.5	55.6	48.5 – 62.7	n.s.
11. Offering you services for preventing diseases (screening, health checks, immunizations)? *	48.7	45.1 – 52.3	41.5	35.5 – 47.5	P = 0.006

**Information and support**					
12. Explaining the purpose of tests and treatments? *	60.2	56.9 – 63.4	68.0	62.8 – 73.2	P = 0.044
13. Telling you what you wanted to know about your symptoms and/or illness? *	60.2	57.0 – 63.4	69.9	65.0 – 74.8	P = 0.005
14. Helping you deal with emotional problems related to your health status?*	49.7	46.6 – 52.8	61.3	55.2 – 67.5	P = 0.004
15. *Helping you understand of following his or her advice?*	51.0	48.1 – 54.0	47.9	41.9 – 53.9	n.s.

**Continuity and cooperation**					
16. *Knowing what s/he had done or told you during earlier contacts?*	53.4	50.0 – 56.9	59.8	52.6 – 67.0	n.s.
17. *Preparing you for what to expect from specialist or hospital care?*	55.7	51.6 – 59.8	56.4	48.3 – 64.5	n.s.

**Facilities availability and accessibility**					
18. *The helpfulness of the staff (other than the doctor)?*	66.1	62.3 – 69.9	72.7	67.4 – 78.0	n.s.
19. *Getting an appointment to suit you?*	1.2	0.6 – 1.8	1.6	0.5 – 2.6	n.s.
20. *Getting through to the practice on telephone?*	72.1	68.7 – 75.4	70.5	65.6 – 75.3	n.s.
21. *Being able to speak to the general practitioner on the telephone?*	58.3	54.4 – 62.1	67.9	61.8 – 74.1	(P = 0.076)
22. *Waiting time in the waiting room?*	38.1	32.4 – 43.7	39.7	31.1 – 48.4	n.s.
23. *Providing quick services for urgent health problems?*	71.6	68.3 – 74.9	76.9	69.9 – 83.9	n.s.

* = significant values (p < 0.05) between CON and AM group

n.s. = difference between CON and AM group not significant

^a ^= 95% Confidence Interval

In addition, more AM patients than CON patients ranked their physicians "excellent" concerning the giving of information and support, helping them to deal with emotional problems related to their health status (61.3% vs. 49.7%, p = 0.004), telling them about what they wanted to know about their symptoms and/or illness (69.9% vs. 60.2%, p = 0.005) and explaining the purpose of tests and treatments (68.0% vs. 60.2%, p = 0.044).

A much higher percentage of the AM patients valued the thoroughness of the GP (70.4% vs. 56.5%, p > 0.001). The patients receiving CON treatment reported that their GPs more frequently provided preventive services, such as screenings, health checks and immunizations (48.7% vs. 41.5%).

## Discussion

It is unlikely that the high patient satisfaction with AM that we found is conveyed by unique factors. Rather, the specific resource-oriented and holistic therapeutic setting of AM is a complex interdependent pattern that positively affects several components of patient satisfaction.

Our findings confirm the results of previous studies that CAM in general [33] and AM in particular [20] lead to high patient satisfaction.

**Figure 1:**
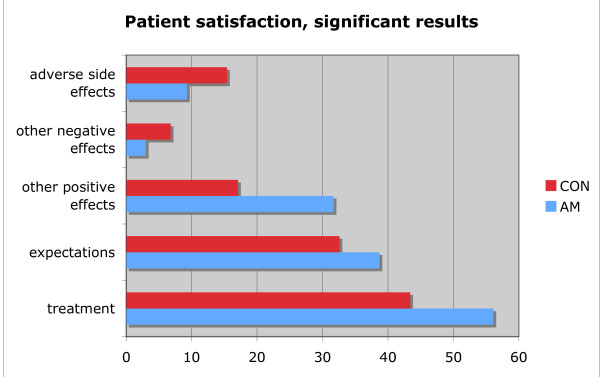
Comparison of significant differences between the AM- and CON-group (in %).

In our study, AM patients show significantly higher treatment satisfaction in all of the five items than CON patients (see figure 1 and table 4). These results are consistent with AM theory, which emphasizes relationship and communication, as well as shared decision-making [12]. The holistic and integrative approach of AM [9,19] would also be expected to be more thorough than a CON approach, since it addresses more potential facets of health and disease [11,34].

### Patients and diagnosis

As in studies investigating CAM [4,33], AM in other countries [12,16,35] and in Switzerland [36,37], urban, middle-aged women (30 to 50 years) with higher education were overrepresented in our AM group (see table 2). Highly educated patients may be better able to follow the AM approach, actively taking part in their treatment. They also might adapt better to stress and changes brought about by the illness, for example through a meaningful support or a positive interpretation of their diseases [14,15]. As AM patients have shown to be more convinced that their lifestyle has an impact on their health [35], these patients with a more active approach in managing their problems may have a greater sense that their condition is manageable and this increases satisfaction [14,18]. AM therapy does not work without the cooperation of patients. Therefore, some AM physicians only accept patients who are highly motivated, responsible and "psychologically mature" enough to work with AM [12]. This inherent selection could explain some differences in the patient groups.

The AM patients in our study, as in prior studies [12,21], suffered more frequently from chronic diseases of the musculoskeletal and respiratory system, mental and behavioural disorders and cancer than CON patients, who suffered more often from diseases of the circulatory system (see table 3).

For these chronic illnesses of our AM patients, as well as for "non life-threatening" diseases, such as psychosomatic or functional/psycho-vegetative disorders or certain pain-syndromes (e.g. migraine) with feelings of ill health, or with marked subjective symptoms for which no severe organic disease is present, further CON diagnostics and treatment were unsatisfactory because of ineffectiveness, adverse effects, or non-compliance [38], or were not indicated.

As chronic illness is the most common cause of disease burden worldwide (often associated with co-morbidity) [4], successful AM treatment could result in a reduction of health care costs [39], in particular, since CAM can lead to improvement or bring relief in the areas of clinical practice in which CON treatment is not fully effective (e.g. musculoskeletal problems, chronic pain, eczema, depression, cancer, etc.) [37]. In patients with these types of common illnesses, CAM methods are often more beneficial, although the cost-effectiveness is disputed [39,40].

### Fulfilment of treatment expectations (see figure 1 and table 4)

A common definition of patient satisfaction is "fulfilment of treatment expectations." Patients choose AM for its holistic and person-centred approach that is tailored to individual needs, or in situations of limited effectiveness of CON in case of chronic diseases and cancer [17,23]. They expect the facilitation of personal learning and development [12], wish to be involved in the management of their illness [41], or want to do everything possible to fight an incurable disease [42].

Further aspects related to the specific AM approach, such as the quality of physician-patient relationship, the use of natural treatments and remedies with few side effects, the activation of self-healing through art therapies, and the wish for the holistic AM therapy [17,23] seem to be key reasons that patients seek AM therapy.

Perhaps some of our AM patients also belong to these "expert patients", who exchanges for the public health system invisible in networks, self helping groups or chartrooms wishing to be involved in the management of their diseases [41].

A growing number of patients, reject the traditional authoritarian and pathologically oriented role of western CON physicians, feel misunderstood, incompletely advised or treated unsatisfactorily. These patients tend to change to CAM methods, which were closely linked to their salutogenitic needs and their expectations to be equal partners with the physicians in treatment decisions [41].

The higher expectation of healing as opposed to relief of symptoms that we found in the AM group (see table 2) may be related to the AM theory that illness is an imbalance among the forces of body, mind and spirit, which can generally be rebalanced or even healed [11]. This may give patients a degree of optimism [14].

### Other effects and adverse side effects (see figure 1 and table 4)

A further positive factor for AM may be significantly fewer adverse side effects. While CON drugs are specifically prescribed for particular physical pathologies and have strong effects and side effects, AM treatments aim to activate the whole person, restore inner balances and activate self-healing capacities at different functional levels [10,12]. This is accomplished by therapies to which the whole person reacts with body, soul and spirit, such as music- or art therapy, eurythmy, or massage. Also, in accordance with its principles of "salutogenesis" [18] and "hygiogenesis" [10,43], AM attempts to specifically activate "life forces", which are considered responsible for all processes of growth, vitality, self-healing, self-regulation, adaptation and regeneration [10]. This is done, as in homeopathy or herbal medicine, through special pharmaceutical preparations from minerals, plants or animal substances (e.g. potentization), aimed at eliciting specific effects. Art therapies and „mild“ agents were known to have only few side effects [10,20] and as such could have contributed to the higher patient satisfaction in the AM group [16,44,45]. This is in line with the observational evidence of high safety and sustainable effects of the treatment with AM on perceived symptoms and to improve quality of life in chronic diseases, including advanced cancer and depression [20].

Further factors that may lead to higher patient satisfaction are the patients' positive attitude towards AM and its theories as well as their expectation or „belief“ of likely benefit. This can be seen as a placebo-response, but underlying this there may also be significant optimism [46] and trust [12,47] of patients who had good experiences with AM or had heard about others who did so, especially in those diseases where CON treatments were at their limits [38]. The fact that AM physicians have the option of prescribing both conventional and anthroposophic therapies might also strengthen trust in AM treatment.

"Other positive effects" in the AM group were perhaps personal experiences with the therapy or factors associated with becoming proactive in their own treatment. Patients may have described a "build up effect" or a "feel good factor" after AM appointments in that patients expressed feeling more positive when they came out than when they went in [12]. This may reflect AM therapy meeting the expected health needs of our patients through a greater focus on individual responsibility and providing deeper-level explanations of health and illness, linking psychological and physical dimensions, which may help to cope with the illness, finding a new meaning of life or self-development [14].

### Other negative effects and more adverse side effects (see figure 1 and table 4)

That CON patients mentioned "other negative effects" and "adverse side effects" more often could reflect a higher risk of side effects or drug interactions with conventional drugs or with drugs taken without knowledge of the physician. Further aspects could be the missing consultation time or that many of the patients' real problems could not be solved by a non-holistic approach.

### Results of the Europep questionnaire

To our knowledge, our study is the first to investigate patient evaluation of their primary care providers comparing AM to CON, using the Europep instrument to provide a subjective assessment of different aspects of care provision in positive and negative terms. The Europep instrument queries judgments by patients, in contrast to satisfaction, which assumed to be a (general) emotional reaction to a specific situation [3]. In international comparisons of Europep results, Swiss patients are known to give high rating scores to their physicians (often the best or second to the best ranking) in items of the dimensions that we classified as "relationship and communication" [3], but these absolute higher ratings in Switzerland would be expected to affect both groups equally and not to bias the comparison of AM and CON in this study.

### Physicians

The structure of our AM practices, which were predominantly situated in the German speaking part of Switzerland, was similar to the structure of other CAM practices [36] and AM practices in other countries [12]. Namely, these CAM and AM practices were more frequently group practices, with more part-time physicians and with less technical equipment than CON practices (table 1). In addition, the CAM and AM practices offered more patient-centred and individualized treatment modalities [40]. The central location of the practices could be explained by the need to serve geographically dispersed patients. As expected, our AM physicians were more likely to speak German.

There has much been written about the setting in which the clinical encounter between a patient and a healthcare professional takes place, which is seen as the core activity of medical care [28,48] and how the physician can contribute to good communication [26]. In the practices of our study, these effective communication and affective relationship dynamics were generally known and certainly implemented, which contributed to the high ranking for both groups. Good communication is particularly important for chronically ill patients, since it improves patient compliance and thus improves the quality of care [49].

### Consultation time

Physicians practicing AM have longer consultations, taking an extended history, addressing constitutional, psychosocial, and biographic aspects of patients' illnesses, and selecting optimal therapy [12]. The consultations lasting seven minutes longer in our AM group seem short when considering the goals and methods of AM as an extension of CON [12]. In that respect and in light of the characteristics of their patients, AM physicians seem to work efficiently, since even CON physicians tend to have longer consultations with chronically ill patients [50].

### Relationship and communication (Questions 1–6, see table 5)

In our study, AM physicians showed higher interest in the personal situation, listened to patients more and involved patients more in decision making than CON physicians.

### Time enough, interest in the patients' personal situation, listening, and making it easy to talk about problems

Our results were supported by results of another study, in which AM patients described their physicians to be good listeners with a 'calm' and 'unrushed' attitude and with a high degree of personal encouragement and interest in their patients [12]. In that study, patients who had employed both AM and CON treatment described the consultation with AM physicians more as a 'dialogue' or 'two way process' compared to the rush consultation with a CON physician [12]. Moreover, our results were consistent with the interpretation that the AM therapeutic concept leads to a more relational and supportive communication style [40].

### Involvement in decisions about medical care

That AM patients in our study more often felt involved in decisions about their medical care than CON patients could have resulted from a key aspect of AM therapy, which is to motivate patients to actively engage in their treatment and to take responsibility for addressing their health problems [12,27]. It is known that shared decision-making is a challenge for both, patients and physicians: patients have to take more responsibility of their treatment, even in case of non-success, and physicians have to respect the patients wishes, even if they decide against the physicians advice. Deciding together improves quality of care [51].

### Physicians' confidentiality

AM patients were more content with data protection than the CON group. This may have positively reinforced (or may simply reflect) their trust in AM physicians.

### Information and support (Questions 12–15, see table 5)

AM physicians in our study explained tests and treatments more often, discussed symptoms and illness more often and helped the patient more often to deal with emotional problems than did CON physicians.

Patients increasingly demand medical advice as well as medical information in a manner and in language that they can understand and increasingly expect that their own concepts of self-healing be incorporated in decisions concerning therapy [41].

### Explaining tests and treatments

Patients externally referred to AM services are particularly impressed with the depth of information covered in consultations [12]. Often it is necessary to inform patients about the approach in AM consultations. To an AM physician, there is no simple catalogue of instruction to treat each particular disease. Rather, AM theory calls for the physician to imagine for each patient "flexible working pictures" implementing the theory of an integrative view of simultaneous interactions of the different subsystems accounted for in the AM understanding of health and illness [9,10]. These pictures intend to help to find the right individual therapy. For the most part, AM practitioners are seen as knowledgeable and flexible in their approach to diagnosis and treatment [12]. AM physicians may give information about the imbalance, which led to the illness and may motivate their patients to participate actively in their treatment.

### Talking about symptoms and illness

Patients with chronic conditions were especially appreciative of the positive approach taken by the AM doctors and a sense of hope they gave that not all possibilities of improving their condition had been exhausted [12]. However, AM physicians tend to give realistic information about there not being any 'guarantees of cure' [12].

### Helping the patient to deal with emotional problems

Chronic or severe illness is often associated with feelings of depression, sadness and anxiety, with philosophical questions of ill health or psychosocial problems accompanying the illness. In this context, emotional problems can become acute. It is helpful to be able to speak to a person of trust about these problems. Through the formulation of the problems in words, the patient can gain a greater objective distance from the problems with which she is concerned. Together with the apparently closer physician-patient relationship of AM patients and the more intensive discussion, these factors could have led the AM patients to reveal more intimate information than did the CON patients. This may have been particularly important, since a higher percentage of AM patients compared to our CON patients suffered from chronic illnesses that required more thorough consultations.

### Medical care (Questions 7–11, see table 5)

Patients expected not only good counselling with time enough to communicate their concerns, to be informed and included in decisions about their illness, or physicians' secrecy, but also services for preventing diseases [3]. In the last category, the CON physicians scored significantly better than AM physicians; but AM physicians were more often judged as being thorough (table 5).

### Services for preventing diseases

The CON practices of our study seem to reflect actual mainstream medicine in Switzerland that offers highly quality technical medicine combined with a personal service, and they also appear to follow current best practices in offering preventive services, such as screenings, health checks and immunizations.

In general, the physical dimension of illness remains the focus of CON. In light of their superior technical equipment, CON practices can perform the necessary diagnostics promptly and are able to diagnose and treat quickly acute health problems, e.g. of the cardiovascular system. This may be one reason that our CON practices treated more patients with cardiovascular diseases than the AM practices.

### Thoroughness

AM patients rated their physicians as more thorough, although they had less technical equipment. This may be due to the longer consultation time with more detailed medical and biographical history-taking and more intensive relationship and communication factors of AM physicians who were experienced in both CON and AM [10,12].

Previous studies showed that patient satisfaction is less related to the therapeutic outcome [52,53], and more to certain aspects of the therapeutic alliance [46,54,55]. Such an alliance presupposes a supportive physician-patient working relationship, in which the physician is seen as helpful, reliable, and successful in achieving common goals [54]. Our results concerning better ratings in "relationship and communication", as well as in "information and support" for the AM physicians, also support this theory. The more time-consuming AM procedure might better fulfil the needs of more critical or expert AM patients, who wish to be informed and take part in medical decisions, especially where the CON treatment is unsatisfactory or at its limit. The AM physician's personality, her empathy, and her willingness to communicate could be decisive factors for the higher patient satisfaction with AM (or even for its effectiveness). AM consultation appears in itself to be a therapeutic intervention working independently or synergistically with the prescribed therapy or agent for this group of patients.

## Limitations

The PEK project was not designed to evaluate the unique aspects of AM.

The inhomogenity and the wide range of patient expectations and the different types of practices with different objectives, strengths and weaknesses, makes it complicated and difficult to assess and compare the two groups [37].

The extent to which out results can be generalized is limited by 1) the low answer rate of CON physicians, 2) the selection of AM physicians (FMH specialist certificate and membership in VAOAS), 3) the disparate return rate of CON and AM patients, 4) older CON patients (older patients tend to be more satisfied), 5) the high percentage of severely ill AM patients (with more negative [56] or paradoxically more positive [57] assessment of satisfaction) 6) the higher educational and socio-demographic level of the AM group, and 7) self-reporting of time by physicians; further, 8) the four week period prior to the follow-up questionnaire being too short to measure long-term satisfaction, and 9) the presumed higher motivation of AM physicians that may have positively influenced patient satisfaction. Alternatively, it may be that our results are skewed from patients previously having had good experience with their physicians.

However, despite their young age, better education (notwithstanding younger and better-educated patients tending to be more critical,) and more severe (as confirmed by the Charlson index) and chronic disease status, our AM patients were more satisfied with their treatment than CON patients.

It can be debated whether to include additional explanatory factors in the statistical models of this study in order to account for potential confounders. Other studies within PEK showed, however, in correspondence with the literature [58], that patients in complementary medicine are characterised by specific motives to seek care and have distinct treatment expectations [44,45].

The analysis of such factors is beyond the scope of a quantitative study within the framework of a health technology assessment to evaluate CAM, and we therefore regarded these factors as intrinsic components of providing and consuming care within CON or AM.

It may be criticized that our data are mainly based on perceived health status and on self-reported subjective assessments of patients instead of objective measures of treatments success. However, patient based assessments of health status have been proven to be valid measures of health in general populations [3,59]. Our study was not aimed at specific treatment procedures but at the health status of a cross-section of AM and CON patients, and therefore we believe that patient satisfaction is a valid measuring tool for our purposes.

## Conclusion

One possible conclusion from the Europep results would be that AM physicians should give more advice on prevention of disease and CON physicians should have longer and perhaps more comprehensive consultations with their patients. Also advisable would be an improved working relationship between AM and CON physicians in which the strengths of both approaches can complement each other, for example in quality circles with interdisciplinary case studies, consultations, liaison projects, or formation of practices or hospitals including CON and AM. This could increase patient satisfaction and thereby improve overall patient care.

Although the pre-post design and the short observation-time of the present as well as the number of limitations from the methods we employed does not allow for confirmative conclusions about comparative outcomes, our findings suggest that AM physicians provide an effective, motivating and satisfying treatment for our self-selected patient population of better educated, female, middle-aged chronically-ill and cancer patients. Our results tend to show that several factors contributed to the higher patient satisfaction and better fulfilment of expectations in the AM group, such as the closer patient-physician relationship in AM, communication in which the patient is more active, the thoroughness and empathy of the physicians, but also the activation of self-healing through art therapies and the use of natural treatments and remedies with few side effects.

Although the cost-benefits of AM even for chronic diseases is disputed, AM seem to be a promising therapy for treating chronic illness and in the areas of clinical practice in which CON treatment is not fully effective. To confirm our results, a more focussed longer-term qualitative study would be necessary.

## Abbreviations

AM: Anthroposophic Medicine; CAM: Complementary and Alternative Medicine; CON: Conventional Medicine; PEK: Programm Evaluation Komplementärmedizin (Complementary Medicine Evaluation Programme); FMH: Foederatio Medicorum Helveticorum (Swiss Medical Association); VAOAS: Vereinigung anthroposophischer Ärzte in der Schweiz (Swiss Medical Association for Anthroposophic Medicine).

## Competing interests

The authors declare that they have no competing interests.

## Authors' contributions

BME wrote the manuscript. FM and PH reviewed and completed the manuscript and provided considerable input with reference to AM and primary care. PH and AB have both held leading positions in the organization of PEK and have actively contributed to the construction of the study protocol and the selection of investigational tools. AB obtained the mandate for the implementation of the project, performed all statistical analyses and completed the manuscript in this context.
